# Can We Do Away With PTBD?

**DOI:** 10.1155/1995/90362

**Published:** 1995

**Authors:** R. D. Bapat, N. N. Rege, R. S. Koti, N. K. Desai, S. A. Dahanukar

**Affiliations:** 1 Department of Pharmacology, Seth G. S. Medical College and K. E. M. Hospital Parel Bombay 400 012 India; 2 Department of Gastroenterology Surgical Services Parel Bombay 400 012 India

## Abstract

Percutaneous Transhepatic Biliary Drainage (PTBD) is performed in surgical jaundice to decompress the biliary tree and improve hepatic functions. However, the risk of sepsis is high in these patients due to immunosuppression and surgical outcome remains poor. This raises a question—can we do away with PTBD? To answer this query a study was carried out in 4 groups of patients bearing in mind the high incidence of sepsis and our earlier studies, which have demonstrated immunotherapeutic potential of Tinospora cordifolia (TC): (A) those undergoing surgery without PTBD (n = 14), (B) those undergoing surgery after PTBD (n = 13). The mortality was 57.14% in Group A as compared to 61.54% in Group B. Serial estimations of bilirubin levels carried out during the course of drainage (3 Wks) revealed a gradual and significant decrease from 12.52 ± 8.3 mg% to 5.85 ± 3.0 mg%. Antipyrine half-life did not change significantly (18.35 ± 4.2 hrs compared to basal values 21.96 ± 3.78 hrs). The phagocytic and intracellular killing (ICK) capacities of PMN remained suppressed (Basal: 22.13 ± 3.68% phago, and 19.1 ± 4.49% ICK; Post drainage: 20 ± 8.48% Phago and 11.15 ± 3.05% ICK). Thus PTBD did not improve the metabolic capacity ofthe liver and mortality was higher due to sepsis. Group (C) patientg received TC during PTBD (n = 16) and Group (D) patients received TC without PTBD (n = 14). A significant improvement in PMN functions occurred by 3 weeks in both groups (30.29 ± 4.68% phago, 30 ± 4.84% ICK in Group C and 30.4 ± 2.99% phago, 27.15 ± 6.19% ICK in Group D). The mortality in Groups C and D was 25% and 14.2% respectively during the preoperative period. There was no mortality after surgery. It appears from this study that host defenses as reflected by PMN functions play an important role in influencing prognosis. Further decompression of the biliary tree by PTBD seems unwarranted.


*HPB Surgery*, 1995, Vol. 9, pp. 5–11
Reprints available directly from the publisher
Photocopying permitted by license only

© 1995 OPA (Overseas Publishers Association)
Amsterdam B.V. Published in The Netherlands
by Harwood Academic Publishers GmbH
Printed in Malaysia

### Can We Do Away With PTBD?

R. D. BAPAT, N. N. REGE*, R. S. KOTI, N. K. DESAI* and S. A. DAHANUKAR*

Department of Gastroenterology Surgical Services and *Department of Pharmacology,
Seth G. S. Medical College and K. E. M. Hospital, Parel, Bombay 400 012, India

(*Received March 25, 1994*)

Percutaneous Transhepatic Biliary Drainage (PTBD) is performed in surgical jaundice to decompress the biliary tree and improve hepatic functions. However, the risk of sepsis is high in these patients due to immunosuppression and surgical outcome remains poor. This raises a question—can we do away with PTBD? To answer this query a study was carried out in 4 groups of patients bearing in mind the high incidence of sepsis and our earlier studies, which have demonstrated immunotherapeutic potential of Tinospora cordifolia (TC): (A) those undergoing surgery without PTBD (n = 14), (B) those undergoing surgery after PTBD (n = 13). The mortality was 57.14% in Group A as compared to 61.54% in Group B. Serial estimations of bilirubin levels carried out during the course of drainage (3 Wks) revealed a gradual and significant decrease from 12.52 ± 8.3 mg% to 5.85 ± 3.0 mg%. Antipyrine half-life did not change significantly (18.35 ± 4.2 hrs compared to basal values 21.96 ± 3.78 hrs). The phagocytic and intracellular killing (ICK) capacities of PMN remained suppressed (Basal: 22.13 ± 3.68% phago. and 19.1 ± 4.49% ICK; Post drainage: 20 ± 8.48% Phago and 11.15 ± 3.05% ICK). Thus PTBD did not improve the metabolic capacity of the liver and mortality was higher due to sepsis. Group (C) patients received TC during PTBD (n = 16) and Group (D) patients received TC without PTBD (n = 14). A significant improvement in PMN functions occurred by 3 weeks in both groups (30.29 ± 4.68% phago, 30 ± 4.84% ICK in Group C and 30.4 ± 2.99% phago, 27.15 ± 6.19% ICK in Group D). The mortality in Groups C and D was 25% and 14.2% respectively during the preoperative period. There was no mortality after surgery. It appears from this study that host defenses as reflected by PMN functions play an important role in influencing prognosis. Further decompression of the biliary tree by PTBD seems unwarranted.

KEY WORDS: PTBD immunosuppression sepsis PMN cells Tinospora cordifolia, host defences.

### INTRODUCTION

The increased risk of surgery in patients with surgical jaundice is well known[1,2,3]. Since the introduction of percutaneous drainage techniques in 1974[4], the initial reports on preoperative external biliary drainage were of dramatic reduction in post-operative mortality[5–12]. Recent trials have however emphasized the complications of drainage techniques thus questioning the advantages of this method[13,14,15]. The hazards of the technique include cholangitis, haemorrhage, biliary leakage, catheter blockage/dislodgement.

The incidence of sepsis following external biliary drainage has been reported to be between 22–33%[17,18,19]. Impairment of function of the immune system in patients with obstructive jaundice has been suggested as the underlying cause for sepsis[19–29]. To control infection, antibiotics have been used, complicating the therapy with development of resistant organisms. Strengthening of host defences is a novel approach which may be complementary to antibiotics to combat infection in patients with obstructive jaundice.

Recently, Tinospora cordifolia, a plant from the traditional Indian system of Medicine (Ayurveda), has

*Address for correspondence:* R. D. Bapat, Professor and Head, Department of Gastroenterology Surgical Services, Seth G. S. Medical College and K. E. M. Hospital, Parel, Bombay-400 012, India.

been shown to improve the surgical outcome in patients with obstructive jaundice by strengthening the host defences[30]. Tinospora cordifolia was administered to patients after institution of external biliary drainage and was continued during the entire drainage period. It is available in the form of pills which contain dried aqueous extract of the stem of the plant. Earlier reports[31,32] have shown its protective effect against a variety of infections and this has been attributed to its immunostimulant property.

The beneficial effect of Tinospora cordifolia in patients undergoing percutaneous drainage prompted us to conduct the present study. In the present study we aimed at evaluating the effects of Tinospora cordifolia in patients with obstructive jaundice undergoing biliary tract surgery, without preoperative Percutaneous Transhepatic Biliary Drainage (PTBD) and compared the results with those undergoing pre operative PTBD and recieving Tinospora cordifolia. The changes in bilirubin, antipyrine half-life and polymorphonuclear functions in terms of phagocytosis and intracellular killing capacity of neutrophils, along with perioperative mortality and morbidity were observed and noted. The efficacy of Tinospora cordifolia was also viewed in the light of changes observed in these parameters in patients who underwent surgery alone or following biliary drainage but did not receive Tinospora cordifolia.

### MATERIALS AND METHODS

A prospective study was carried out with the approval of the Hospital Ethics committee, in 57 consecutive patients with suspected malignant extrahepatic biliary tract obstruction. The diagnosis was confirmed by ultrasonography, percutaneous transhepatic cholangiography and/or ERCP. The final proof of malignant disease was obtained by peroperative biopsy (or autopsy in patients who died preoperatively). The patients with disseminated malignancy, hepatocellular carcinoma, patients presenting with complications, those with other associated diseases, pregnant women and those taking other traditional drugs/remedies were excluded from the study. Malignant lesions included carcinoma of head of pancreas (n = 27), ampullary carcinoma (n = 16), carcinoma gall bladder (n = 10), cholangiocarcinoma (n = 2) and metastases at porta hepatis (n = 2).

There were 37 men and 20 women who were included in the study. The age range was 17 to 73 yrs; median age being 48.5 yrs. The median weight for the group was 42.5 kg. The median duration of illness was 45 days. Written, informed consent was obtained from each patient before randomly allocating them into 4 groups.

**Group A:** Patients directly underwent surgery without preoperative PTBD (n = 14).

**Group B:** Patients underwent PTBD preoperatively for 3 weeks, followed by surgery (n = 13).

**Group C:** Patients underwent PTBD and received treatment with Tinospora cordifolia preoperatively for 3 weeks, followed by surgery (n = 16).

**Group D:** Patients received treatment with Tinospora cordifolia only, pre-operatively for 3 weeks, followed by surgery (n = 14).

Tinospora cordifolia was used in the form of pills prepared from dried aqueous extract of the stem of the plant; (1 pill = 65 mg). The dose selected was 16 mg/kg/day in 3 divided dose and was extrapolated from previous animal experiments[26].

All patients received Vitamin K and perioperative antibiotics. Biliary enteric bypass surgery was done in all cases.

At the time of admission a profile of liver function tests, renal chemistry and haemogram was obtained. Metabolic function of the liver was assessed by determining the half-life of elimination of antipyrine[34]. 10 ml of venous blood was collected in a sterile heparinized tube for determination of phagocytic and killing capacities of neutrophils[30]. These values assessed on admitting the patients were considered as basal values. In the groups undergoing PTBD (Group B and C), in addition to above tests, bile was collected for culture and antibiotic sensitivity at the time of insertion of drain, weekly during the period of drainage and intra-operatively. Weekly assessment was carried out by determining – plasma bilirubin (total and direct), antipyrine half-life (t 1/2)[34,37] and phagocytic and killing activity of neutrophils[30]. Pre and postoperative mortality and morbidity were noted.

### RESULTS

All the four groups were matched with respect to clinical features, demographic data and parameters assessed at the time of admission (basal values).

**Group A:** The basal plasma bilirubin levels were 12.9 ± 6.4 mg% total bilirubin and 7.2 ± 10.1 mg% direct

**Table 1** Changes in total and direct bilirubin, antipyrine half-life over a period of 3 weeks in patients with obstructive jaundice.

| | Total Bilirubin (mg%) | | | | Direct Bilirubin (mg%) | | | | Antipyrine t1/2 (hrs) | | | |
|---|---|---|---|---|---|---|---|---|---|---|---|---|
| Weeks | 0 | 1 | 2 | 3 | 0 | 1 | 2 | 3 | 0 | 1 | 2 | 3 |
| Group A (n = 14) | 12.9 ±6.4 | 13.63 ±6.1 | 14.12 ±5.2 | NS 14.19 ±5.4 | 7.2 ±10.1 | 8.0 ±5.3 | 8.34 ±2.3 | NS 8.61 ±1.41 | 21.23 ±5.6 | 22.36 ±6.3 | 24.7 ±5.4 | NS 25.8 ±6.4 |
| Group B (n = 13) | 12.52 ±8.37 | 8.1 ±5.3 | 6.9 ±3.4 | 5.85* ±3.0 | 7 ±4.2 | 5.29 ±3.9 | 4.48 ±3.0 | 3.6* ±2.1 | 21.96 ±3.78 | 20.65 ±2.1 | 19.14 ±3.7 | NS 18.35 ±4.2 |
| Group C (n = 16) | 9.4 ±2.68 | 7.2 ±1.6 | 5.2 ±2.3 | 4.43* ±2.1 | 6.96 ±2.19 | 5.34 ±1 | 3.38* ±1.68 | 3.5* ±2.4 | 19.49 ±3.14 | 22.2 ±2.85 | 21.88 ±8.74 | NS 17.13 ±0.18 |
| Group D (n = 14) | 10.06 ±3.4 | 9.43 ±4.3 | 8.2 ±2.4 | NS 8.48 ±2.82 | 7.4 ±2.87 | 6.4 ±3.67 | 5.32 ±2.17 | NS 5.9 ±2.02 | 23.23 ±3.9 | 21.65 ±2.8 | 20.18 ±3.43 | NS 19.38 ±4.46 |

Paired 't' test; p < 0.05; NS = not significant.

bilirubin. The basal antipyrine half-life was 21.23 ± 5.6 hours, which was found to be significantly prolonged as compared to normal values (AP t1/2 = 14–16 hrs). The polymorphonuclear functions in terms of % phagocytosis and % intracellular killing capacity of neutrophils were 21.23 ± 5.1% and 17.87 ± 4.5% respectively. These values were significantly lower then those established for 30 normal healthy individuals (30.4 ± 5.1% phago, P<0.01 and 26.41 ± 4.4% ICK; p<0.05). Bilirubin estimation and antipyrine t1/2 determined preopera-tively were as follows: total bilirubin 14.19 ± 5.4 mg%; direct bilirubin 8.61 ± 1.41 mg% and Ap t1/2 25.8 ± 6.4 hrs. The perioperative mortality in this group was 57.14% (8/14). Of these 5 patients died of liver cell failure, 2 of septicaemia and 1 of biliary peritonitis which developed secondary to anastomotic leak.

**Group B:** In patients who underwent preoperative PTBD there was a significant decrease in the bilirubin levels at the end of 3 weeks of drainage. The total and direct plasma bilirubin levels decreased to 5.85 ± 3 mg% and 3.6 ± 2.1 mg% respectively from initial values of 12.52 ± 8.3 mg% and 7 ± 4.2 mg% (P<0.05). However the antipyrine half-life did not show any significant change inspite of 3 weeks of drainage (Pre-drainage value: 21.96 ± 3.78 hrs and 3 weeks post-drainage value: 18.35 ± 4.2 hrs). The basal phagocytic and killing capacities of neutrophils in these patients were found to be depressed as compared to normal values (22.13 ± 3.68% phago and 19.1 ± 4.49% ICK; P<0.05). A further suppression of these functions was observed during the drainage period; 20 ± 8.48% phago and 11.15 ± 3.05% ICK (P<0.05).

Complications seen during the drainage period were substantial. These were, catheter blockage in 2 patients, cholangitis in 2 patients, bile leak in peritoneal cavity due to kinking of catheter between abdominal wall and liver in 1 patient, mild renal failure which was reversible in 1 patient, pneumonia in 2 patients and pleural effusion in 1 patient. The perioperative mortality was 61.54% (8/13). 3 patients died of liver cell failure, 4 patients of septicaemia and 1 patient of acute renal failure.

**Group C:** As was seen in Group B, patients from this group who also underwent preoperative PTBD showed a significant decrease in bilirubin levels. At the end of 3 weeks of drainage total bilirubin decreased from 9.4 ± 2.68 mg% to 4.43 ± 2.1 mg% and direct bilirubin from 6.96 ± 2.19 mg% to 3.5 ± 2.4 mg%. However, antipyrine half-life did not show a significant change (basal value: 19.49 ± 3.14 hrs and 3 weeks post drainage value: 17.13 ± 0.18 hrs). The basal values of % phagocytosis and % intracellular killing capacity of neutrophils were significantly lower than normal values (19.4 ± 7.07% phago and 18.61 ± 8.79% ICK). However at the end of 3 weeks, a significant rise in both the functions was observed (30.29 ± 4.68% phago and 30 ± 4.84% ICK). These values were comparable to normal values of these functions.

Three patients developed wound infection, 1 had GI haemorrhage and 1 patient developed pneumonia. The perioperative mortality was 25% (4/16). One patient died of bile peritonitis following catheter breakage. Repositioning was with great difficulty. Post-mortem revealed multiple intra-abdominal abscesses.

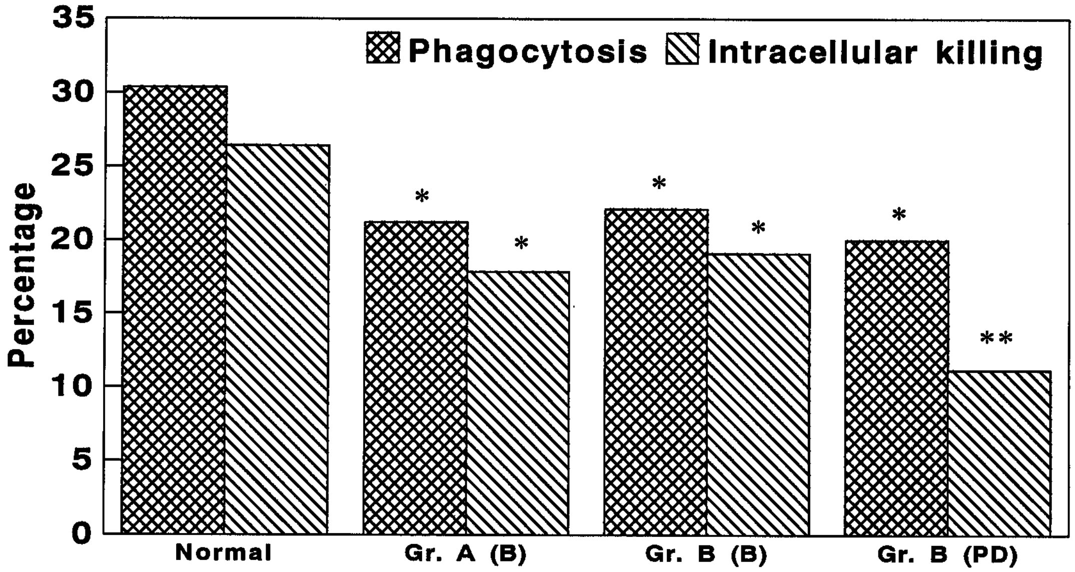

PD : Post drainage ** p < 0.05
Gr. B (B) vs (PD)
B : * p < 0.05 N vs (B)

**Figure 1** Effect of PTCD on PMN function (Group B) PMN functions.

Two patients died of septicaemia of which 1 had severe GI haemorrhage and bleeding from the drain. One patient died of liver cell failure.

**Group D:** The patients who received only Tinospora cordifolia therapy during preoperative period, showed a decrease in bilirubin levels at the end of 3 weeks of treatment (Total bilirubin: from 10.06 ± 3.4 mg% to 8.48 ± 2.82 mg% and direct bilirubin: from 7.4 ± 2.87 mg% to 5.9 ± 2.02 mg%) but the difference was not significant. Antipyrine levels did not show any significant change (Basal: 23.23 ± 3.9 hrs and post Tinospora cordifolia therapy: 19.38 ± 4.46 hrs).

The basal % phagocytosis and % intracellular killing capacity were significantly depressed (22.72 ± 6.25% Phago and 18.36 ± 4.25% ICK) as compared to normal values. At the end of 3 weeks of treatment with Tinospora cordifolia both functions showed a dramatic rise to near normal levels (30.4 ± 2.99% Phago and 27.51 ± 6.19% ICK).

One patient developed wound infection and 1 patient had pneumonia which were treated effectively. The perioperative mortality was 14.2% (2/14). One patient died of septicaemia and the other of liver cell failure.

### DISCUSSION

Biliary decompression and curative/palliative surgery are performed in obstructive jaundice. However these procedures are associated with considerable morbidity and mortality. The main complications are sepsis, renal failure, haemorrhage and impaired wound healing[35,36]. These have been associated with deranged hepatic function, portal and systemic endotoxaemia[36].

The deranged hepatic function was considered the underlying mechanism responsible for various complications in jaundiced patients. Preoperative biliary drainage therefore provided a logical solution to prevent postoperative complications. Biliary decompression was thought to allow the hepatocytes to regain their functional capacity. This approach was followed enthusiastically and the initial reports showed a reduction in postoperative mortality from 28% to 8%[6]. Recent prospective studies[13,14,15] however do not confirm the benefits of preoperative biliary drainage. The hazards of this technique outweigh the possible advantages. Thus there appear to be two schools of thought 1. to carry out surgery taking the risk of postoperative complications 2. to operate after PTBD.

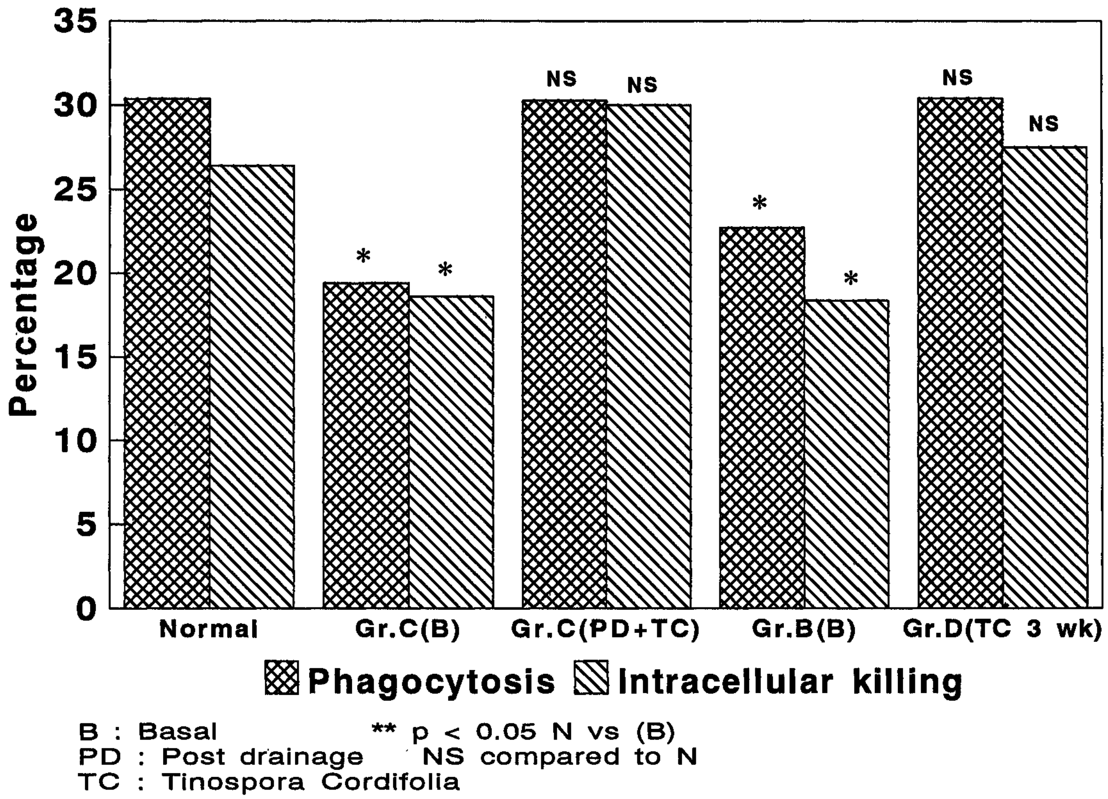

**Figure 2** Effect of TC on PMN function (Group C & D) PMN functions.

In the present study in group A where patients underwent surgery without preoperative drainage the mortality was 57.14%. The high levels of Bilirubin and prolonged half-life of antipyrine indicated deranged hepatic function. Deaths due to septicemia indicate poor host defences. Indeed all patients showed suppression of polymorphonuclear function in terms of phagocytic and intracellular killing capacities of neutrophils (Fig. 1).

In group B where patients underwent preoperative drainage there was a significant fall in bilirubin levels over a period of 3 weeks of drainage. Often the plasma bilirubin level is taken as a guideline for improvement in hepatic function and the patient is taken up for surgery when bilirubin levels decrease. However as seen in our study, the reduction in bilirubin level is not accompanied by improvement in half-life of antipyrine. Even though the bilirubin levels were halved, antipyrine half-life, an indicator of metabolic function of liver remained prolonged (Table 1). This indicates that esti-mation of bilirubin levels alone will not give a true measure of the functional capacity of the liver. Such a patient if operated would still carry a high risk of postoperative complications. This fact has also been stressed by McPherson *et al.*[37]. The most dreaded complication after PTBD is sepsis. In this group (B) bile was sterile at the time of insertion of drain but the culture positivity increased with increasing duration of drainage, and 11 of 13 patients showed bactobilia during the drainage period despite strict aseptic precautions and antibiotics. 4 out of 13 patients died in septicemia. In all these patients the basal PMN functions were depressed. It is noteworthy that after PTBD there is further suppression of phagocytosis and intracellular killing capacity of neutrophils, which is possible due to secondary sepsis (Fig. 1). Sepsis is also the limiting factor which prevents optimal drainage till the liver functions return to normal which may well exceed 3 weeks. In spite of decompressing the biliary system by PTBD liver functions did not improve and the majority of patients (7/8) died from septicemia (4/7) and liver cell failure (3/7). The drainage associated complications were multiple and significant.

Addition of an immunostimulant to the therapeutic armamentarium (Group C) was found to improve PMN functions (Fig. 2). Even though the duration of PTBD remained the same as in Group B, there was a fall in post operative mortality (25% in Group C VS 61.54% in Group B). The mortality following septicaemia was lowered (12.5% in Group C VS 30.76% in Group B). The antipyrine half-life remained virtually unaltered. The relation between improvement of host defences and survival cannot be explained from this study. In our earlier study[26] in patients with obstructive jaundice due to malignant lesions, an improved survival in the group which received Tinospora corditolia (92% sur-vival VS 40% survival in patients not receiving the drug) was associated with bolstering of depressed PMN functions. Similarly the reduction both in post-operative mortality and that following septicaemia may be attributed to the enhanced PMN function.

The question which was to be answered was whether we can do away with preoperative external biliary drainage and treat the patients only with an immunostimulant during the preoperative management of the jaundiced patient. In Group D patients were treated for 3 weeks with Tinospora cordifolia following which they underwent surgery without preoperative PTBD. During this period there was no significant rise in the bilirubin levels and half-life of antipyrine (Table 1). This cannot be explained conclusively, however a hepatoprotective effect of Tinospora cordi-folia has been evaluated in various models of liver diseases[32,33]. The PMN functions after 3 weeks of Tinospora cordifolia therapy have risen to near normal levels (Fig. 2). This group showed the least overall mortality (14.2%) and also the lowest mortality from septicaemia (7.15%).

There appears to be no advantage associated with external biliary drainage before surgery in obstructive jaundice. It is associated with many complications and there is no improvement in metabolic function of the liver. On the contrary there is further suppression of host defences which will compound the problems faced in obstructive jaundice.

Non specific host defences appear to be the most important prognostic factor. Immunomodulators such as Tinospora cordifolia reduce the risk involved and improve the outcome after surgery in obstructive jaundice and should be included in the preoperative management of the jaundiced patients. Immunomodulation is the need of the hour and PTBD should be avoided to prevent sepsis and immunosuppression.

### ACKNOWLEDGEMENTS

We would like to thank Ms. Mala Kulkarni; Ms. Vidya Nayak; Ms. Neela Rajwade for extending technical assistance.

### REFERENCES

1. Wait, R. B. and Kahng, K. U. (1989) Renal failure complicating obstructive jaundice. *Am. J. Surg.*, **157**, 256–263.
2. Clairmont, P. and Haberer, V. H. (1911) Ueber Anurie nach Gallensteinoperationen. *Mitt Grenzgeb Med Chir.*, **22**, 159–172.
3. Whipple A. O., Parsons, W. B. and Mullins, C. R. (1935) Treatment of carcinoma of the ampulla of vater. *Ann. Surg.*, **102**, 763–779.
4. Molnar W. and Stockum, A. E. (1974) Relief of obstructive jaundice through percutaneous transhepatic catheter a new therapeutic method. *AJR*, **122**, 365–367.
5. Takada, T., Hanyu, F., Kobayashi, S. and Uchida, Y. (1976) Percutaneous transhepatic cholangial drainage: direct approach under fluoroscopic control. *J. Surg. Oncol.*, **8**, 83–97.
6. Nakayama, T., Ikeda, A. and Okuda, K. (1978) Percutaneous transhepatic drainage of the biliary tract. Technic and results in 104 cases. *Gastroenterology*, **74**, 554–559.
7. Gundry, S. R., Strodel, W. E., Knol, J. A., Eckhauser, E. and Thompson, N. W. (1984) Efficacy of preoperative biliary tract decompression in patients with obstructive jaundice. *Arch. Surg.*, **119**, 703–708.
8. Norlander, A., Kalin, B. and Sundblad, R. (1982) Effect of percutaneous transhepatic drainage upon liver function and postoperative mortality. *Surg. Gynecol. Obstet.*, **155**, 161–166.
9. Ellison, E. C., Van Aman, M. E. and Carely, L. C. (1984) Preoperative transhepatic biliary decompression in pancreatic and periampullary cancer. *World Surg.*, **8**, 862–871.
10. Gobien, R. P., Stanley, J. H., Soucek, C. D., Anderson, M. C., Vujic, I. and Gobien, B. S. (1984) Routine preoperative biliary drainage: effect on management of obstructive jaundice. *Radiology*, **152**, 353–356.
11. Denning, D. A., Ellison, E. C. and Carey, L. C. (1981) Preoperative percutaneous transhepatic biliary decompression lowers operative morbidity in patients with obstructive jaundice. *Am. J. Surg.*, **141**, 61–65.
12. Gouma, D. J., Wesdrop, R. I. C., Oostenbroek, R. J., Soeters, P. B. and Greep, J. M. (1983) Percutaneous transhepatic drainage and insertion of an endoprosthesis for obstructive jaundice. *Am. J. Surg.*, **145**, 763–767.
13. Hatfield, A. R. W., Terblanche, J., Fataar, S., Kernoff, L., Tobias, R., Girdwood, A. H., Harries-Jones, R. and Marks, I. N. (1982) Preoperative external biliary drainage in obstructive jaundice. A prospective controlled clinical trial. *Lancet*, **II**, 896–899.
14. Pitt, H. A., Gomes, A. S., Lois, J. F., Mann, L. L., Deutsch, L. S. and Longmire, W. P. (1985) Does preoperative percutaneous biliary drainage reduce operative risk or increase hospital cost? *Ann. Surg.*, **201**, 545–553.
15. McPherson, G. A. D., Benjamin, I. S., Hodgson, H. J. F., Bowley, N. B., Allison, D. J. and Blumgart, L. H. (1984) Preoperative percutaneous transhepatic biliary drainage: the results of a controlled clinical trial. *Br. J. Surg.*, **71**, 371–375.
16. Gouma, D. J. and Moody, F. G. (1984) Preoperative percutaneous biliary drainage: use or abuse. *Surg. Gastroenterol.*, **3**, 74–80.
17. Mueller, P. R. and Harbin, W. P. (1980) Percutaneous transhepatic biliary drainage: technique, results and applications. *Radiology*, **135**, 1–3.

18. Mcpherson, G. A. D., Benjamin, I. S., Habib, N. A., Bowley, N. B. and Blumgart, L. H. (1982) Percutaneous transhepatic drainage in obstructive jaundice: advantages and problems. *Br. J. Surg.*, **69**, 261–4.
19. Blenkharn, J. I., McPherson, G. A. D. and Blumgart, L. H. (1984) Septic complications of percutaneous transhepatic biliary drainage. Evaluation of a new closed drainage system. *Am. J. Surg.*, **147**, 318–321.
20. Holman, J. M., Rikkers, L. F. and Moody, F. G. (1979) Sepsis in the management of complicated biliary disorders. *Am. J. Surg.*, **138**, 809–813.
21. Vane, D. W., Redlich, P., Weber, T., Leapman, S., Siddiqui, A. R. and Grosfeld, J. L. (1988) Impaired immune function in obstructive jaundice. *J. Surg. Res.*, **45**, 287–293.
22. Maggiore, G., De Giacomo, C., Scotta, M. S., Siena, S., Maccario, R. and Vitiello, A. (1982) Cell—mediated immunity in children with chronic cholestasis. *J. Pediatr. Gastroenterol. Nutr.*, **1**, 385–388.
23. Gianni, L., Di Padova, F., Zuin, M. and Podda, M. (1980) Bile acid-induced inhibition of the lymphoproliferative response to phytohemagglutin and pokeweed mitogen: An in vitro study. *Gastroenterology*, **78**, 231–235.
24. Keane, R. M., Gadacz, T. R., Munster, A. M., Birmingham, W. and Winchurch, R. A. (1984) Impairment of human lymmphocyte function by bile salts. *Surgery*, **95**, 439–443.
25. Roughneen, P. T., Didlake, R., Kumar, S. C., Kahan, B. D. and Rowlands, B. J. (1987) Enhancement of heterotopic cardiac allograft survival by experimental biliary ligation. *Transplantation*, **43**, 437–438.
26. Rege, N. N., Nazareth, H. M., Bapat, R. D. and Dahanukar, S. A. (1989) Modulation of immunosuppression in obstructive jaundice by Tinospora cordifolia. *Indian J. Med. Res.*, **90**, 478–483.
27. Drivas, G., James, O. and Wardle, N. (1976) Study of reticuloendothelial phagocytic capacity in patients with cholestasis. *Br. Med., J.*, **1**, 156–159.
28. Pain, J. A. (1987) Reticulo-endothelial function in obstructive jaundice. *Br. J. Surg.*, **74**, 1091–1094.
29. Newberry, W. M., Shorey, J. W., Sanford, J. P. and Combes, B. (1973) Depression of lymphocyte reactivity to phytohemagglutin by serum from patients with liver disease. *Cell Immunol.*, **6**, 87–97.
30. Rege, N., Bapat, R. D., Koti, R., Desai, N. K. and Dohanukar, S. A. (1993) Immunotherapy with Tinospora cordifolia. A new lead in the management of obstructive jaundice. *Indian J. Gastroenterol*, Vol. 12, No. **1**, 5–8.
31. Thatte, U. M. and Dahanukar, S. A. (1989) Immuno therapeutic modification of experimental infections by Indian Medical Plants. *Phytotherapy Res.*, **3**, 43–49.
32. Rege, N. N., Dahanukar, S. A. and Karandikar, S. M. (1984) Hepatoprotective effect of Tinospora Cordifolia against carbon tetrachloride induced liver damage. *Indian Drugs*, **21**, 544–555.
33. Malgi, S. P. (1989) Modulation of liver injury by plant products. *Thesis*. (Msc).
34. Nazareth, H. M., Rege, N. N., Nayak, V. K., Desai, N. K., Someshwar, V., Dahanukar, S. A. and Bapat, R. D. (1990) Antipyrine clearance: Prognostic marker for obstructive jaundice. *Indian J. Gastroenterol.*, **9**(1), 63–66.
35. Greve, J. W. M. Complications in Obstructive jaundice: an experimental study on etiology and prevention: Thesis.
36. Diamond, T. and Rowlands, B. J. (1991) Endotoxaemia in obstructive Jandice. *HPB Surg.*, **4**(2), 81–94.
37. McPherson, G. A. D., Benjamin, I. S., Boobis, A. R. and Blumgart, L. H. (1985) Antipyrine Elimination in patients with obstructive jaundice A predictor of outcome. *Am. J. Surg.* **149**, 140–143.

